# Saline versus contrast-enhanced ultrasound for confirmation of intranodal needle position: reply to Fung et al.

**DOI:** 10.1007/s00247-023-05800-9

**Published:** 2023-11-11

**Authors:** Julia Wagenpfeil, Claus Christian Pieper

**Affiliations:** Division for Minimally-Invasive Lymph Vessel Therapy, Department of Diagnostic and Interventional Radiology, University Hospital of Bonn, Venusberg-Campus 1, 53127 Bonn, Germany

Dear Editors,

We read with interest the article by Fung et al. on the feasibility of contrast-enhanced ultrasound (CEUS) to confirm intranodal needle position for dynamic contrast-enhanced magnetic resonance lymphangiography (DCMRL) in children [1]. The authors describe their experience with CEUS for this application in seven children with bilateral inguinal nodal access (14 punctured nodes). Included patients had a median age of 13 years (interquartile range 3.5–7.5 years) with attempted punctures of lymph nodes larger than 2 mm in diameter. A cannulation success rate of 12/14 nodes (85.7%) was reported.

Over the last decade, the armamentarium of lymphatic imaging and interventional treatment options has grown rapidly and has expanded to include applications in very young patients [2]. In this respect, DCMRL plays an important role in the diagnostic work-up of patients with suspected lymphatic abnormalities but remains a technique with high logistical and technical demands. The most important factor for a successful DCMRL examination is stable nodal needle position used for contrast injection during the investigation [2, 3]. Confirmation of adequate needle position prior to the actual MR examination is therefore of utmost importance to avoid unnecessary patient transfers and unacceptably long examination times.

Initial reports described confirmation by injection of water-soluble radiographic contrast-agent under fluoroscopy [1, 2]. However, as this technique requires a combined Angio-MR-(XMR)-suite, its application is limited. Nadolski et al. subsequently published their initial experiences with needle position validation by CEUS in adults forgoing the need for an XMR-suite [3]. In their report, CEUS demonstrated the need for needle repositioning in 6/28 cases (21.4%). Fung et al. now demonstrate the feasibility of this approach in pediatric patients [1]. However, although CEUS seems to be a viable tool for needle position conformation for DCMRL, the question remains whether the additional off-label ultrasound contrast application is really necessary—especially in children.

The largest published series (171 punctured lymph nodes) on technical success of nodal DCMRL demonstrated that the use of saline solution rather than ultrasound contrast agent is sufficient for needle position verification [4]. In this cohort, overall technical success was observed in 169/171 lymph node punctures (98.8%). Primarily venous run-off was observed in only 6/171 lymph nodes (3.5%) on DCMRL and was resolved by minimal needle retraction in the scanner. Although this report focused on adults, 16 children (<10 years: *n*=11 and <2 years: *n*=5) with lymph node diameters as low as 1 mm were also included. DCMRL was successful in this pediatric subgroup of patients using only saline solution for needle position validation.

In conclusion, although CEUS may be helpful in some cases of nodal DCMRL, we advocate needle position verification by saline solution only as this technique yields a very high success rate without the need for additional off-label ultrasound contrast-agent administration in children. However, all recommendations in children are currently based on small case series (*n*=7 [1] and *n*=16 [4]) so that further studies with larger patient cohorts are necessary to reach more meaningful conclusions regarding optimal puncture procedures.
